# Tumor Necrosis Factor-related Apoptosis-inducing Ligand (TRAIL) Signaling and Cell Death in the Immature Central Nervous System after Hypoxia-Ischemia and Inflammation*

**DOI:** 10.1074/jbc.M113.512350

**Published:** 2014-02-07

**Authors:** Anton Kichev, Catherine I. Rousset, Ana A. Baburamani, Steven W. Levison, Teresa L. Wood, Pierre Gressens, Claire Thornton, Henrik Hagberg

**Affiliations:** From the ‡ Centre for the Developing Brain, Perinatal Brain Injury Group, Kings College London, London SE1 7EH, United Kingdom,; the § Perinatal Center, Institutes of Clinical Sciences and Neuroscience and Physiology, The Sahlgrenska Academy, University of Gothenburg, 405 30 Gothenburg, Sweden,; the ¶ Department of Neurology and Neuroscience, New Jersey Medical School, Rutgers University, Newark, New Jersey 07103-1709, and; ‖ Inserm, U676, F-75019 Paris, France

**Keywords:** Cell Death, Hypoxia, Neuroinflammation, Neurons, Oligodendrocytes, Trail, Immature CNS

## Abstract

Tumor necrosis factor-related apoptosis-inducing ligand (TRAIL) is a member of the TNF family. The interaction of TRAIL with death receptor 4 (DR4) and DR5 can trigger apoptotic cell death. The aim of this study was to investigate the role of TRAIL signaling in neonatal hypoxia-ischemia (HI). Using a neonatal mouse model of HI, mRNA, and protein expression of TRAIL, DR5 and the TRAIL decoy receptors osteoprotegerin (OPG), mDcTRAILR1, and mDcTRAILR2 were determined. *In vitro*, mRNA expression of these genes was measured in primary neurons and oligodendrocyte progenitor cells (OPCs) after inflammatory cytokine (TNF-α/IFN-γ) treatment and/or oxygen and glucose deprivation (OGD). The toxicity of these various paradigms was also measured. The expression of TRAIL, DR5, OPG, and mDcTRAILR2 was significantly increased after HI. *In vitro*, inflammatory cytokines and OGD treatment significantly induced mRNAs for TRAIL, DR5, OPG, and mDcTRAILR2 in primary neurons and of TRAIL and OPG in OPCs. TRAIL protein was expressed primarily in microglia and astroglia, whereas DR5 co-localized with neurons and OPCs *in vivo*. OGD enhanced TNF-α/IFN-γ toxicity in both neuronal and OPC cultures. Recombinant TRAIL exerted toxicity alone or in combination with OGD and TNF-α/IFN-γ in primary neurons but not in OPC cultures. The marked increases in the expression of TRAIL and its receptors after cytokine exposure and OGD in primary neurons and OPCs were similar to those found in our animal model of neonatal HI. The toxicity of TRAIL in primary neurons suggests that TRAIL signaling participates in neonatal brain injury after inflammation and HI.

## Teaser

**Background:** TRAIL induces apoptosis through interaction with receptor DR5.

**Results:** Animal model of neonatal hypoxia-ischemia (HI) and primary cultures subjected to oxygen glucose deprivation show increased expression of TRAIL and DR5; TRAIL is toxic for primary neurons.

**Conclusion:** TRAIL-mediated cell death contributes to brain injury after HI.

**Significance:** TRAIL signaling pathways may represent a valid drug target for protection against neonatal HI.

## Introduction

Hypoxia-ischemia (HI)^2^ is an important cause of perinatal brain injury both in term infants suffering from intrapartum asphyxia and in preterm infants exposed to hypotensive events (1, 2). The pathophysiological mechanisms are complex and processes such as apoptosis, necroptosis, mitochondrial impairment, oxidative stress, and inflammation are involved (3). There are several lines of evidence that inflammation induces cell death after HI (4). However, we lack the molecular understanding of how immuno-inflammatory mediators provoke death of neurons and oligodendroglial precursor cells (OPCs) in neonatal HI. During inflammation, both infiltrating immune cells and resident CNS glial cells produce reactive oxygen species, release excitatory amino acid agonists, proinflammatory cytokines (*e.g.* IL-1α, IL-18, TNF-α), chemokines (5, 6), and tumor necrosis factors (*e.g.* TNF-α, TNF-β, FasL, TRAIL, TWEAK) (5, 7–9). We hypothesized that tumor necrosis factor superfamily members participate in the evolution of injury after HI because several ligand-receptor pairs belonging to the TNF family have been implicated in the injurious cascade (10).

Previous work has implicated the TNF-α cluster (11) and Fas signaling (7, 12, 13) in the cellular loss after HI, but little is known about the role of TRAIL signaling in the development of neonatal brain injury. In humans, four membrane-bound and one soluble receptor for TRAIL have been identified. Of these, two contain cytoplasmic death domains (DR4 and DR5) and have the capacity to induce apoptotic cell death (14, 15), whereas DcR1 (TRAIL-R3), DcR2 (TRAIL-R4), and the soluble osteoprotegerin (OPG) lack functional death domains and are considered to function as decoy receptors (16–19). Similar receptors for TRAIL exist in rodents. There are two membrane-spanning decoy receptors, mDcTRAILR1 and mDcTRAILR2 (20), one soluble OPG (19), and only one death-mediating TRAIL receptor, which has the highest homology with the human DR5 receptor (21).

TRAIL-DR4/5 can induce apoptosis via Fas-associated protein with death domain/death-inducing signaling complex (FADD/DISC)/caspase-8 signaling in several cell types, including neurons and oligodendroglia (10). This pathway is important in the pathogenesis of adult stroke (22), trauma (23), infection (24), and multiple sclerosis (25), but there is limited information available with respect to the involvement of TRAIL and its receptors in the demise of immature neurons such as in neonatal HI (26).

The aim of the present study is to explore the expression and the cellular distribution of TRAIL and its binding receptors in an animal model of neonatal HI; second, to investigate the induction of TRAIL molecules *in vitro* in primary neurons and OPCs after oxygen glucose deprivation (OGD) and/or TNF-α/IFN-γ administration; and third, to evaluate the cellular toxicity of TRAIL in these cells.

## EXPERIMENTAL PROCEDURES

### 

#### 

##### Neonatal Hypoxia-ischemia

C57BL/6 wild-type mice were obtained from Moellegaard Breeding and Research Center A/S (Skensved, Denmark). Neonatal HI was induced at postnatal day 9 according to methods described by Rice *et al.* (27) but modified for mice (28, 29). Mice of both sexes were anesthetized with isoflurane (3% for induction and 1.5% for maintenance) in nitrous oxide/oxygen (1:1). The left common carotid artery was ligated with PROLENE suture #6-0 (ETHICON). After the surgical procedure, the wounds were closed and infiltrated with a local anesthetic. After 1 or 2 h of recovery with the dam, the pups were placed in a chamber perfused with a humidified gas mixture (10% oxygen in nitrogen) for 60 min at 36 °C. The animals were kept in humidified air at 36 °C for extra 10 min before and after the hypoxic exposure. After the hypoxia, the pups were returned to their dams. This procedure results in brain injury in the ipsilateral hemisphere consisting of cerebral infarction and selective neuronal death in the cortex, striatum, hippocampus, and the thalamus, leaving the contralateral hemisphere undamaged. Control littermates were neither operated on nor subjected to hypoxia. The Animal Ethical Committee of Gothenburg approved all animal experiments (no. 269/01). Pups were killed by decapitation at 2, 8, 24, and 72 h after HI and controls at postnatal day 9 and postnatal day 12 (*n* = 5 at each time point). Brains were removed and rapidly frozen on dry ice. Cortex, hippocampus, thalamus, subcortical white matter, and striatum were microdissected from each hemisphere in a frozen state.

##### Primary Cell Culture

Cell culture medium was purchased from Invitrogen or Sigma. FBS and all cell culture medium supplements were purchased from Sigma unless otherwise noted. Recombinant human FGF-2 and recombinant mouse or rat TNF-α and IFN-γ were purchased from Peprotech. Recombinant mouse TRAIL was purchased from R&D Systems. TRAIL was denatured by incubating at 95 °C for 5 min followed by rapid cooling to 4 °C.

##### Primary Neurons

Primary cortical neurons were prepared from C57BL/6 embryonic day 15 mice as described previously (30). Briefly, cortices from embryos in a single litter were dissected, meninges were removed, and tissue was pooled. Cortices were roughly chopped before incubation in 0.25% trypsin/EDTA followed by trituration. Cells were pelleted by centrifugation, resuspended, and plated in Neurobasal medium supplemented with 1× B27 (Invitrogen), 1× streptomycin/amphotericin B (Invitrogen), and 300 μm glutamine. Treatments were carried out on mature neurons grown for a minimum of 12 days *in vitro*.

##### Primary OPC Culture

OPCs were prepared from newborn Sprague-Dawley rats as described previously (31). In brief, forebrain cortices were removed from postnatal day 0–2 rat pups and dissected. Tissues were enzymatically digested with DNase I and 0.25% trypsin and then mechanically dissociated. Cells were resuspended in minimum essential Eagle's medium (Sigma) supplemented with 10% FBS, 2 mm l-glutamine (Sigma), 100 μg/ml penicillin-streptomycin solution (Sigma), and 0.6% glucose and plated in T75 flasks at a density of 2 × 10^5^/cm^2^. Mixed glial cell cultures were grown for 11 days, and OPCs were purified using the shaking method (32). In brief, the mixed glial cell culture was shaken for 1.5 h at 260 rpm to remove microglia, and remaining cells were shaken for 18h to detach the OPCs from astrocytes. Purified OPCs were seeded onto poly-d-lysine-coated 60-mm or 24-multiwell plates at a density of 3 × 10^4^/cm^2^ in N2S medium. N2S medium consists of 66% DMEM/F-12 supplemented with (0.66 mg/ml BSA, 10 ng/ml d-biotin, 5 μg/ml insulin, 20 nm progesterone, 100 μm putrescine, 5 ng/ml selenium, 50 μg/ml apo-transferrin, 100 μg/ml penicillin, 100 μg/ml penicillin-streptomycin solution, and 0.5% FBS) and 34% of the same medium conditioned by the B104 neuroblastoma cell line for 3 days, supplemented with 5 ng/ml FGF-2 (Preprotech), and 0.5% FBS. OPCs were amplified for 6 days before experiments were performed.

##### OGD and Treatment of Primary Cells

OGD was induced by replacing the cell medium with artificial cerebrospinal fluid and incubating the cells in a modular incubator chamber MIC-101 (Billups-Rothenberg, Inc.) filled with a gas mixture of 5% CO_2_ and 95% N_2_. Cells plated in 24-multiwell dishes (used for cell death experiments) were subjected to 1 h of OGD, whereas cells plated in 60-mm dishes (used for RNA extraction) were subjected to 4 h of OGD. After OGD, the artificial cerebrospinal fluid was replaced with fresh medium for OPC and with 50% new:50% preconditioned medium for the neurons. The medium in the control cells was replaced correspondingly.

We optimized the OGD duration for the primary mouse neurons by conducting time course studies and optimized cytokine concentrations by performing dose-response studies. Those studies established that 4 h of OGD induced ∼50% cell death in the neuronal cultures plated in p60 dishes (data not shown). Neurons plated in 24-well plates were much more sensitive to OGD; therefore, we used 1 h for our cell death studies. Cytokines were evaluated at concentrations from 1 ng/ml to 500 ng/ml. None of them displayed toxicity toward neurons; therefore, for our studies we chose 200 ng/ml as we found no difference in the expression levels of studied proteins between 200 and 500 ng/ml (data not shown).

Treatments with recombinant inflammatory cytokines were performed after the OGD with final concentrations of 200 ng/ml mouse TNF-α and 200 ng/ml mouse IFN-γ for neurons and 200 ng/ml rat TNF-α and 200 ng/ml rat IFN-γ for OPCs. Treatment with 1 μg/ml recombinant mouse TRAIL was performed 16 h after the OGD/cytokine treatment. The concentration was chosen after dose response study as the lowest toxic concentration for neurons (data not shown). All recombinant proteins were dissolved in PBS.

##### RNA Isolation

Total RNA from mouse brains was isolated using the RNeasy mini kit (Qiagen) according to the manufacturer's instructions. The RNA was quantified by spectrophotometry at 260 nm, and the OD was determined by the 260/280 ratio. The OD of the RNA was between 1.9 and 2.1, and the quality of the RNA was further evaluated by electrophoresis on a 1.1% agarose, 2.2 mol/liter formaldehyde gel to ascertain that there was no degradation. Six micrograms of total RNA from neocortex, hippocampus, thalamus, and striatum was thereafter pooled to represent a gray matter sample for each hemisphere. The same RNA isolation procedure and quality check was performed for RNA isolated from cell cultures.

##### Quantitative RT-PCR

The reverse transcription reaction was performed using a High Capacity cDNA reverse transcription kit (Applied Biosystems) according to the manufacturer's instructions. Quantitative RT-PCR experiments were performed using the StepOnePlus^TM^ real-time PCR systems, TaqMan probes, and TaqMan Gene Expression Master Mix (Applied Biosystems). All reactions were conducted in duplicate and corrected to GAPDH expression. Data were analyzed using the delta threshold cycle (*C_T_*) method.

##### Western Blot

30 μg of total protein lysate per sample was separated by electrophoresis using 10% Bis-Tris NuPAGE® Novex gels (Invitrogen) and XCell SureLock® Mini-Cell (Invitrogen). Proteins were transferred to polyvinylidene fluoride membranes using an iBlot® gel transfer device (Invitrogen). Membranes were blocked with 5% nonfat dry milk in Tris-buffered saline containing 0.1% Tween 20 (TBS-T) and immunoblotted overnight at 4 °C with anti-TRAIL antibody (R&D Systems) or anti-DR5 antibody (R&D Systems) diluted 1:2500 in TBS-T. After washing with TBS-T, membranes were incubated for 1 h with HRP-conjugated anti-goat antibody at room temperature. Membranes were washed with TBS-T and developed with SuperSignal West Pico chemiluminescent substrate (Thermo Life Science). The images were taken with ImageQuant^TM^ LAS4000 digital imaging system (GE Healthcare).

##### Fluorescent Immunohistochemistry

Paraffin-embedded brain sections were deparaffinized with xylene and ethanol and pretreated with heating for 20 min in 10 mm citric acid, pH 6.0, with 0.1% Tween 20. Sections were blocked for 20 min with 5% horse serum, 1% BSA in PBS before incubation with primary antibodies overnight at 4 °C. We used goat anti-TRAIL and anti-DR5 antibody (R&D Systems) mouse anti-glial fibrillary acidic protein (GFAP) (Sigma) rabbit anti-Olig2 (Millipore), Cy3-conjugated NeuN (Millipore), and biotinylated Lycopersicon esculentum lectin (Vector). For visualization we used Alexa Fluor 488-conjugated anti-goat antibody, Alexa Fluor 546 anti-mouse antibody, and Alexa Fluor 546 streptavidin (Invitrogen). Sections were analyzed using a Leica DM6000 B fluorescent microscope and a Leica TCS SP5 confocal microscope. Images were processed using Leica LAS AF lite and ImageJ software. The intensity of the TRAIL and DR5 staining in particular cell type was assessed for every region of interest (cells with positive staining for cell type specific marker) and compared with the overall intensity in the rest of the cells in the image (cells negative for that particular marker).

##### Assessing Cell Viability with Methylthiazolyldiphenyl-tetrazolium Bromide Assay

Methylthiazolyldiphenyl-tetrazolium bromide was dissolved in PBS and added to cell medium at a final concentration of 500 μg/ml. After a 30-min (neurons) or 1-h (OPC) incubation, the medium was removed, and the purple formazan crystals produced by cells were dissolved in dimethyl sulfoxide. Absorbance was measured at 590 nm on a spectrophotometer.

##### Assessing Cell Viability by Counting the Total Number of Viable Cells

Cells were plated on poly-d-lysine-coated chamber slides (NUNC). After the treatments the cells were stained with anti-cleaved caspase-3 antibody (Cell Signaling) and DAPI. The total number of caspase-3 negative nuclei for every condition was counted using a Leica DM6000 B fluorescent microscope.

##### Statistical Analysis

Statistical analysis was performed using Prism GraphPad 5.0 software (Graph-Pad Software). Data are expressed as means ± S.E. Comparisons between the experimental groups were made using one-way analysis of variance followed by the Dunnett's (when comparing treatment conditions *versus* control) or Tukey's (when comparing the treatments *versus* control and *versus* another treatment) post-test or using two-way analysis of variance followed by Bonferroni post test, as appropriate.

## RESULTS

### 

#### 

##### Neonatal HI Increases the Expression Levels of TRAIL and TRAIL Receptors

**FIGURE 1.**
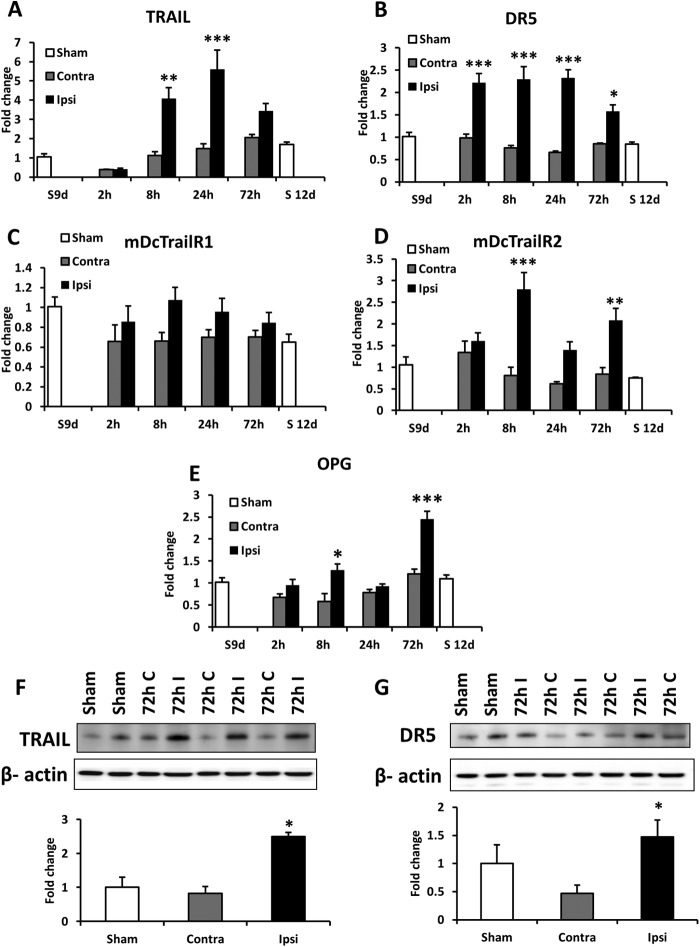
Effects of HI on the expression levels of TRAIL (*A*), DR5 (*B*), mDcTrailR1 (*C*), mDcTrailR2 (*D*), and OPG (*E*) in neonatal mice are shown. mRNA levels were measured in 9- and 12-day-old sham-operated animals (*white bars*), contralateral (*Contra*; *gray bars*), and ipsilateral (*Ipsi*; *black bars*) hemispheres of the brain 2, 8, 24, and 72 h after the insult. The results are expressed as fold change compared with 9-day-old sham-operated animals for every gene. Western blot detection of TRAIL (*F*) and DR5 (*G*) protein in sham-operated (*Sham*, *white bars*), ipsilateral (*I*, *gray bars*), and contralateral hemisphere (*C*, *black bars*) brain lysates 72 h after HI. *Error bars* represent average ± S.E. *, *p* < 0.05; **, *p* < 0.01; ***, *p* < 0.001 *versus* corresponding contralateral side (*n* = 5 (*A–E*) and *n* = 3 (*F* and *G*)).

The expression of TRAIL and TRAIL receptors DR5, OPG, mDcTrailR1, and mDcTrailR2 were evaluated in a neonatal mouse model of unilateral HI. There was a trend (not statistically significant) of decreasing TRAIL expression in both hemispheres 2 h after insult, followed by a >3-fold increase in TRAIL mRNA levels in the ipsilateral compared with the contralateral side of the brain at 8 and 24 h after HI (Fig. 1*A*). The expression of mRNA for DR5 was significantly induced at 2, 8, 24, and 72 h after HI (Fig. 1*B*). In addition, the mRNA expression for mDcTrailR2 and OPG were both increased at 8 and 72 h after the insult (Fig. 1, *D* and *E*), whereas the levels of mDcTrailR1 remained unchanged (Fig. 1*C*). There were no significant differences in expression of any of the genes between the contralateral side of the brain (exposed to hypoxia) and sham-operated, normoxic animals.

**FIGURE 2.**
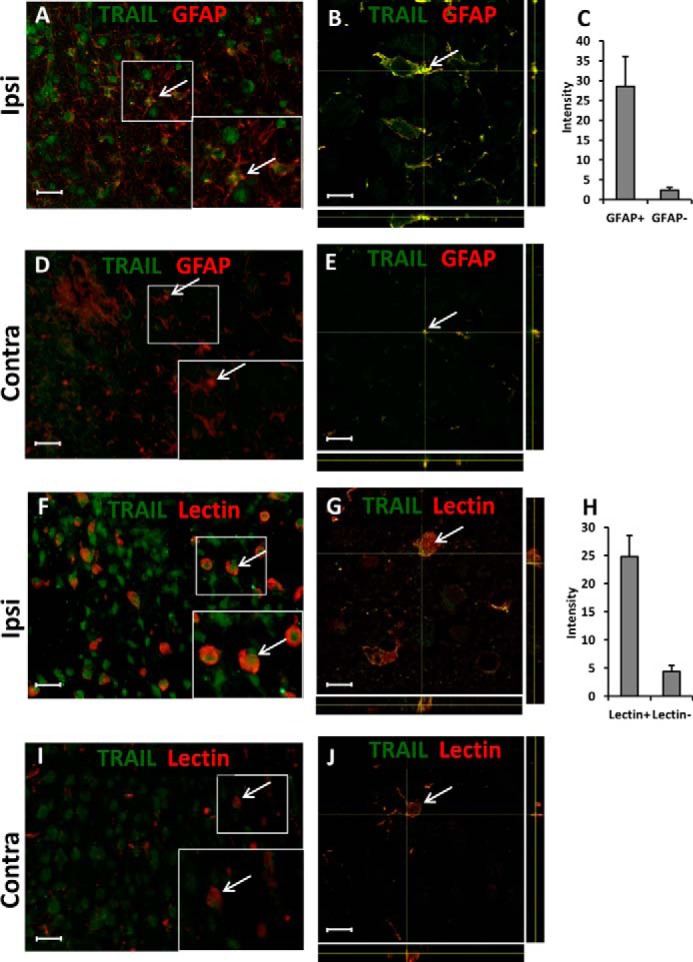
Representative images of conventional (*A*, *D*, *F*, and *I*) and confocal (*B*, *E*, *G*, and *J*) fluorescence microscopy show TRAIL (*green*) immunolabeling in paraffin sections from ipsilateral (*Ipsi*; *A*, *B*, *F*, and *G*) and contralateral (*Contra*; *D*, *E*, *I*, and *J*) hemispheres of the mouse brain at 72 h after HI. There was TRAIL immunoreactivity in GFAP-positive (*red*) astroglia (*A–E*) and lectin-positive (*red*) microglia (*F–J*). GFAP- and lectin-positive cells exhibited stronger TRAIL immunostaining than the GFAP- and lectin-negative cells (*C* and *H*). *Scale bar*, 30 μm (*A*, *D*, *F*, and *I*) and 10 μm (*B*, *E*, *G*, and *J*). Typical GFAP- or lectin-positive cells are indicated by *arrows*.

**FIGURE 3.**
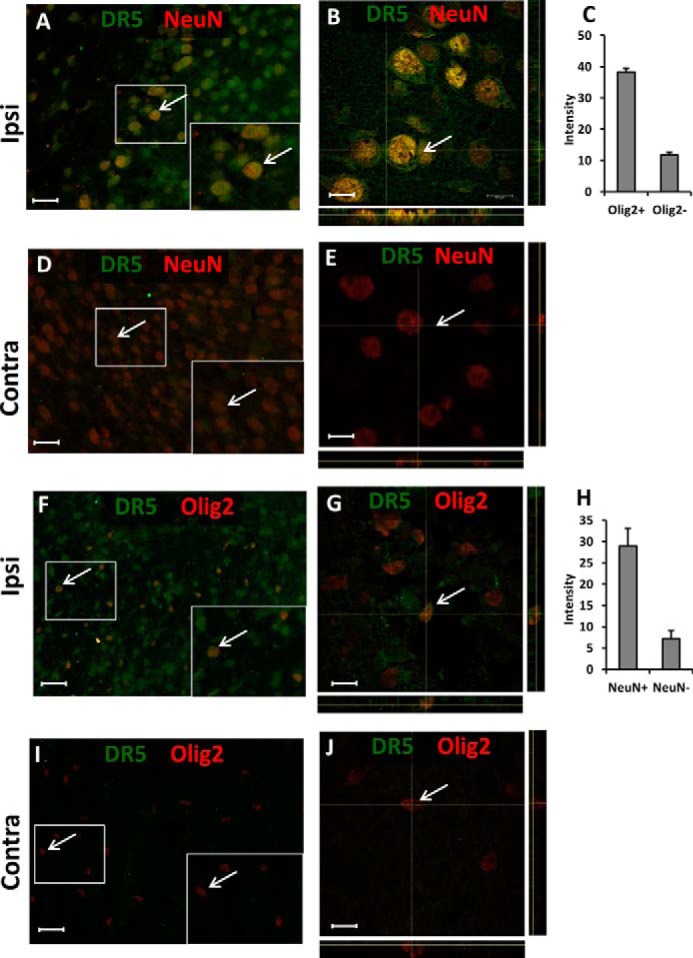
**Conventional (*A*, *D*, *F*, and *I*) and confocal (*B*, *E*, *G*, and *J*) fluorescence microscopy images demonstrating DR5 (*green*) immunostaining in NeuN-positive (*red*) neurons (*A–E*) and Olig2-positive (*red*) oligodendroglial cells (*F–J*) in the brain at 72 h after HI in neonatal mice.** The DR5 immunostaining was more pronounced in the ipsilateral (*Ipsi*) hemisphere (*A*, *B*, *F*, *G*) compared with the non-injured contralateral (*Contra*; *D*, *E*, *I*, and *J*) hemisphere. NeuN- and Olig2-positive cells immunostained more intensely for TRAIL than the NeuN- and Olig2-negative cells (*C* and *H*). *Scale bar*, 30 μm (*A*, *D*, *F*, and *I*) and 10 μm (*B*, *E*, *G*, and *J*). Typical Olig2- or NeuN-positive cells are indicated by *arrows*.

Protein expression was determined at 72 h post insult to allow earlier mRNA changes to be translated and accumulated within the cell. As might be expected from the mRNA analysis, TRAIL and DR5 protein expression was also increased after HI (Fig. 1, *F* and *G*). To investigate which cells were responsible for the enhanced expression of TRAIL/DR5, we performed immunofluorescence on brain sections 72 h after HI. For both proteins, ipsilateral expression was much stronger than contralateral expression confirming the association of increased production of TRAIL, DR5, and brain injury (Fig. 2 and see Fig. 3).

TRAIL immunofluorescence was strongest in GFAP-positive astrocytes (Fig. 2, *A–E*) and in lectin-positive microglia (Fig. 2, *F–J*), whereas low levels of expression were observed also in NeuN-positive neurons and in Olig2-positive cells (data not shown). Thus, we can consider that astrocytes, microglia, and, to a minor extent, neurons and OPCs accounted together for all of the TRAIL immunoreactivity. To more rigorously establish that the astrocytes are predominant sources of TRAIL in the injured brain, we assessed the intensity of TRAIL staining in GFAP-positive cells compared with TRAIL staining in GFAP-negative cells (Fig. 2*C*). Similar measurements were performed for microglial cells using the cell-specific marker Lycopersicon esculentum lectin (Fig. 2*H*). Our measurements showed 12-fold higher TRAIL staining in GFAP-positive cells and 5-fold higher in lectin-positive cells compared with other brain cells.

In contrast, DR5 was expressed predominantly in neurons (Fig. 3, *A–E*) and to some extent in OPCs (Fig. 3, *F–J*) 72 h after HI. Our measurements showed 4-fold higher DR5 staining in the NeuN-positive (Fig. 3*C*) and 3-fold higher staining in Olig2-positive cells (Fig. 3*H*) compared with other brain cells in the ipsilateral side of the brain. As OPCs and neurons are vulnerable and severely compromised after HI, the increase of DR5 expression in these cells implicates TRAIL-DR5 signaling in neonatal brain injury.

##### OGD and Inflammation Increase the Expression of TRAIL and TRAIL Receptors in Primary Neurons and OPCs

To gain new insights into TRAIL-mediated cell death, we investigated TRAIL and its receptors in primary mouse neurons and primary rat OPCs *in vitro*. These cells were exposed to either OGD and/or proinflammatory cytokines (TNF-α and IFN-γ) to mimic HI and the neuroinflammation relevant to perinatal brain injury (5, 33).

##### Primary Neurons

**FIGURE 4.**
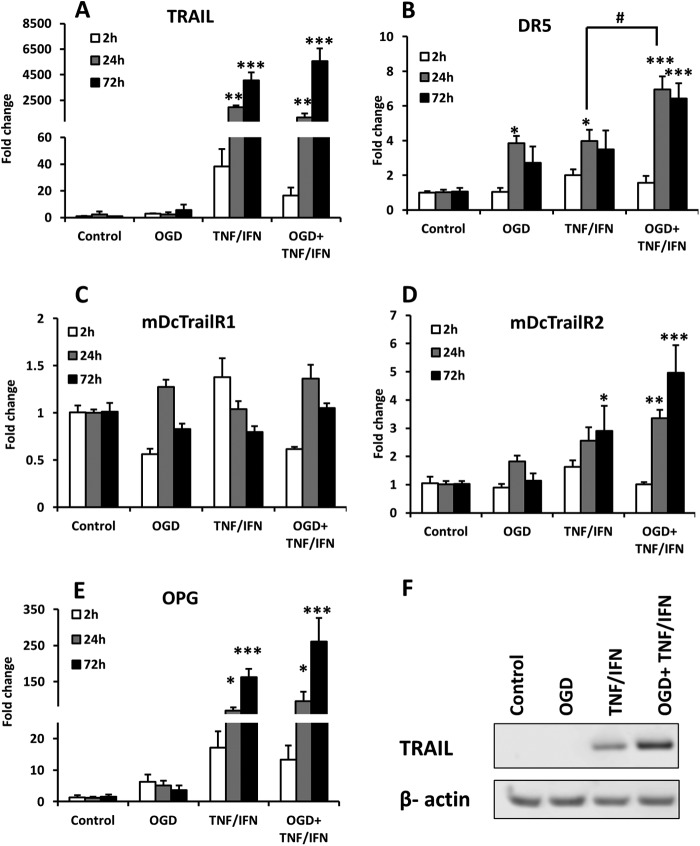
Shown is the effect of OGD and IFN-γ/TNF-α treatments on expression levels of TRAIL (*A*), DR5 (*B*), mDcTrailR1 (*C*), mDcTrailR2 (*D*), and OPG (*E*) in primary neurons. mRNA levels were measured at 2 h (*white bars*), 24 h (*gray bars*), and 72 h (*black bars*) after exposure. The results are expressed as fold change compared with non-treated control cells. *Error bars* represent mean ± S.E., *, *p* < 0.05; **, *p* < 0.01; ***, *p* < 0.001; #, *p* < 0.05 *versus* corresponding controls (*n* = 4). TRAIL expression levels were confirmed at protein level by Western blot (*F*).

Co-administration of TNF-α and IFN-γ induced an increase of mRNA for TRAIL after 24 and 72 h of exposure, whereas OGD had no or little effect on TRAIL expression alone or in combination with the cytokines (Fig. 4*A*). The changes were confirmed at protein level by Western blot 72 h after the treatment (Fig. 4*F*). The mRNA expression of DR5 was significantly and equally induced at 24 h by both OGD and inflammatory cytokines, and the combination of OGD and cytokines evoked an additive increase for DR5 expression at 24 and 72 h (Fig. 4*B*). The mRNA for mDcTrailR2 was significantly but moderately induced by TNF-α/IFN-γ at 72 h (Fig. 4*D*). The expression of OPG was substantially increased by the cytokines treatment as well (Fig. 4*E*). The combination of OGD + TNF-α/IFN-γ showed a tendency to increase the expression of both OPG and mDcTrailR2 even further but the difference was not significant compared with TNF-α/IFN-γ treatment alone (Fig. 4, *D* and *E*). The expression of mDcTrailR1 was not affected by any of these conditions (Fig. 4*C*).

##### Primary OPCs

**FIGURE 5.**
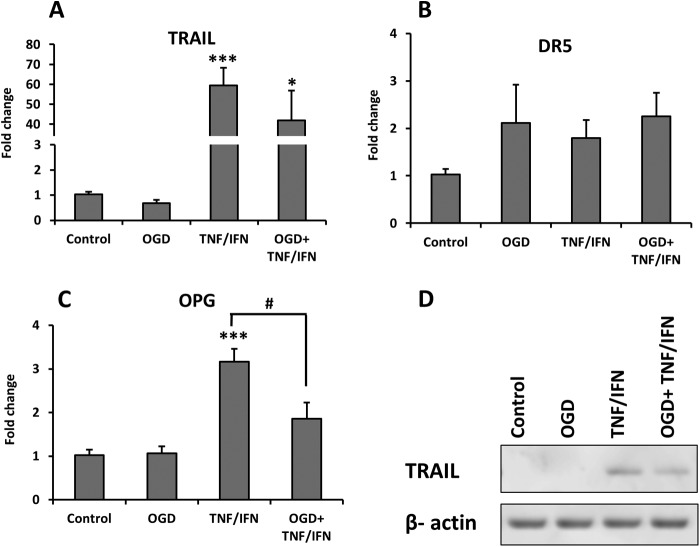
Shown is the effect of OGD and IFN-γ/TNF-α exposure on expression levels of TRAIL (*A*), DR5 (*B*), and OPG (*C*) in primary OPCs. mRNA levels were measured with quantitative RT-PCR 48 h after the treatment. The results are expressed as fold change compared with non-treated control cells. *Error bars* represent mean ± S.E.*, *p* < 0.05; ***, *p* < 0.001 *versus* corresponding controls (*n* = 5). TRAIL expression levels were confirmed at protein level by Western blot (*D*).

**FIGURE 6.**
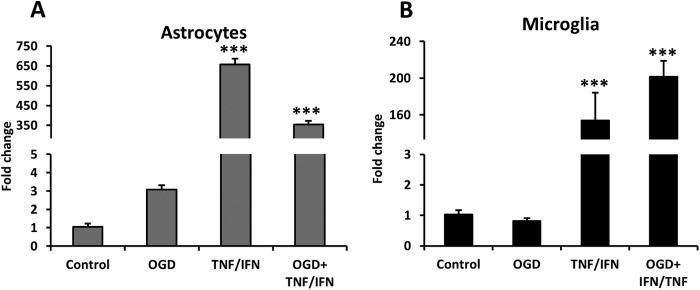
**Effect of OGD and IFN-γ/TNF-α treatments on expression levels of TRAIL in primary astrocytes (*A*) and microglia (*B*).** mRNA levels were measured at 48 h after exposure. The results are expressed as fold change compared with non-treated control cells. *Error bars* represent mean ± S.E. ***, *p* < 0.001; #, *p* < 0.05 *versus* corresponding controls (*n* = 5).

TNF-α/IFN-γ exposure induced a marked increase of TRAIL mRNA and protein (Fig. 5, *A* and *D*). Mean DR5 mRNA levels were slightly higher after both OGD and TNF-α/IFN-γ, but these increases were not statistically significant (Fig. 5*B*). In addition, there was a moderate elevation of OPG mRNA expression (Fig. 5*C*). OGD did not affect TRAIL (Fig. 5*A*) and OPG (Fig. 5*C*); in fact, we observed a decrease in their expression when OGD was combined with the proinflammatory cytokines, reaching significance when compared with TNF-α/IFN-γ alone in the case of OPG (Fig. 5*C*). In addition, we assessed the expression of TRAIL in the primary rat astrocytes (Fig. 6*A*) and microglia (Fig. 6*B*) and found significant increases in expression of TRAIL in both cell types after TNF-α and IFN-γ treatment, confirming the immunohistochemistry data from the animal model.

##### Recombinant TRAIL Is Toxic for Primary Neurons but Not for Primary OPCs

**FIGURE 7.**
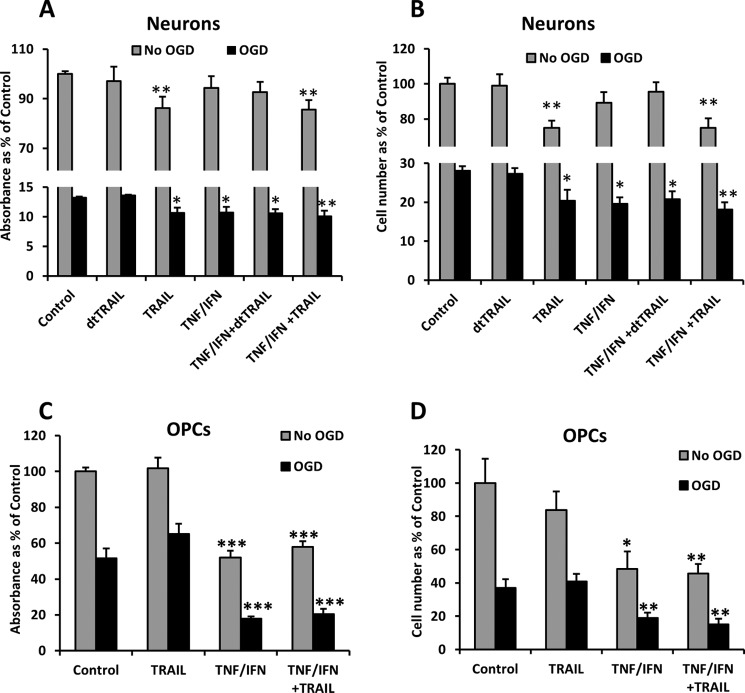
**Effect of OGD, IFN-γ/TNF-α, TRAIL, and dtTRAIL treatment on cell survival measured by methylthiazolyldiphenyl-tetrazolium bromide (*A* and *C*) and counting cleaved caspase-3-negative cells (*B* and *D*) 48 h after the insult.** Primary neurons treated with IFN-γ/TNF-α, TRAIL, or dtTRAIL and combination or subjected to OGD and treated with IFN-γ/TNF-α, TRAIL, or dtTRAIL and a combination (*A* and *B*). Primary OPC treated with IFN-γ/TNF-α, TRAIL, or dtTRAIL and a combination or subjected to OGD and treated with IFN-γ/TNF-α, TRAIL, or dtTRAIL and a combination (*C* and *D*). The results are expressed as percent change of non-treated control cells. *Error bars* represent mean ± S.E. Statistical significance was calculated by comparing every treatment *versus* control. *, *p* < 0.05; **, *p* < 0.01; ***, *p* < 0.001 *versus* corresponding controls.

To determine the toxicity of TRAIL, primary cells were treated with recombinant TRAIL. Cell viability was evaluated by methylthiazolyldiphenyl-tetrazolium bromide assay (Fig. 7, *A* and *C*) and by counting cleaved caspase-3-negative nuclei (Fig. 7, *B* and *D*) 48 h after the exposure. In addition, TRAIL treatment was combined with OGD and inflammatory cytokine exposure to assess possible interactions. Recombinant TRAIL decreased neuronal viability to 80% by 48 h of exposure, and its toxicity was not aggravated further when combined with TNF-α/IFN-γ. The proinflammatory cytokine treatment alone had no toxic effects on the neurons. OGD significantly reduced neuronal survival compared with control, and addition of TRAIL further enhanced OGD-induced neuronal death (Fig. 7, *A* and *B*). Proinflammatory cytokines exerted similar toxicity under OGD conditions but did not further exacerbate the neuronal death induced by OGD+TRAIL (Fig. 7, *A* and *B*). We verified the specificity of TRAIL toxicity by treating the cells with denatured TRAIL (dtTRAIL). The treatment with dtTRAIL did not induce any toxicity alone or in combination with OGD or TNF-α/IFN-γ compared with its corresponding control. In summary, TRAIL exerted significant toxicity under all conditions, supporting TRAIL participation in the development of neuronal death after HI.

In primary OPC cultures, TNF-α/IFN-γ and OGD induced ∼50% cell death. Co-exposure with cytokines and OGD increased injury further as compared with either insult alone with up to 80% of the OPCs undergoing cell death (Fig. 7, *C* and *D*). When compared with neurons, OPCs appear to be equally sensitive to OGD but more sensitive to TNF-α/IFN-γ. Contrary to our expectations, TRAIL was not toxic when administered alone, and it did not modulate OPC cell death induced by cytokines or OGD (Fig. 7, *C* and *D*).

## DISCUSSION

In this study, we have shown for the first time that expression of TRAIL and the TRAIL receptor DR5 are markedly induced in the neonatal brain after HI *in vivo* and, interestingly, that these changes are accompanied by overexpression of mDcTrailR2 and OPG. TRAIL was predominantly expressed by microglia and astroglia in agreement with previous reports in the adult brain after ischemia (22) but was also detected in neurons and OPCs. DR5 was expressed primarily on neurons and OPCs, *i.e.* in cells highly vulnerable to HI insults (34). These *in vivo* data suggested that TRAIL is produced and released by activated glial cells whereupon it could exert toxic effects on OPCs and neurons, unless TRAIL is neutralized by OPG and mDcTrailR2. Qualitatively, the changes in expression of TRAIL, DR5, OPG, and mDcTrailR2 in neuronal cultures were similar to changes observed *in vivo*.

Another important finding from our studies is that the TNF-α/IFN-γ treatment more potently induced TRAIL and OPG than OGD in both neuronal and OPC cultures. In contrast, DR5 was overexpressed after OGD in neurons, and the levels were further enhanced if OGD was combined with cytokine administration. DR5 was poorly expressed in OPC cultures and was not induced by any of the *in vitro* exposures (Fig. 5*B*). Furthermore, in OPCs, we observed that OGD additive treatment significantly attenuated the OPG up-regulation caused by TNF-α/IFN-γ treatment alone (Fig. 5*C*). It has been reported that mature oligodendrocytes hardly expressed TRAIL death receptors but that they were markedly up-regulated in active multiple sclerosis lesions (25) and that the expression of TRAIL death receptors can be provoked by p53 overexpression (35). However, no studies to date have evaluated the influence of OGD and cytokine treatment on OPC TRAIL receptor expression.

We found that recombinant TRAIL treatment is toxic for neurons but not for OPCs. Furthermore, the susceptibility of neurons to TRAIL was not further enhanced by co-exposure to cytokines and/or OGD. This could be explained by the pronounced increase of mDcTrailR2 and especially of OPG as both could bind TRAIL and limit its toxicity on DR5 (18, 20). Similarly, cytokine treatment was not toxic to neurons despite increasing the expression of both TRAIL and DR5. We suspect that the increased expression of the decoy receptors OPG and mDcTrailR2 after cytokine treatment prevented TRAIL toxicity. After OGD, however, the TNF-α/IFN-γ treatment was toxic for the neurons. This finding corroborates animal models in which the combination of HI with inflammation (LPS treatment) increases the severity of the injury (36) and with *in vitro* studies showing increased TNF-α toxicity after OGD (37).

In our OPC experiments, TRAIL exposure did not lead to or increase the vulnerability of the OPCs to any of the conditions. The TNF-α/IFN-γ treatment, however, proved to be highly toxic to OPCs, which confirms previously published data (38). More importantly, OGD exacerbated this effect as the toxicity of OGD + cytokine treatment compared with OGD alone was significantly higher than the toxic effect of cytokine treatment compared with non-treated controls (Fig. 7, *C* and *D*). This finding once again agrees with animal models (36, 39) and shows for the first time the additive toxic effect of OGD and cytokine treatment on OPCs.

The lack of toxicity of TRAIL in OPCs alone, or in combination with OGD/cytokines, is best explained by the fact that DR5 is poorly expressed and not induced significantly by these stimuli in OPC cultures. Alternatively, the high levels of OPG, especially after TNF-α/IFN-γ exposure could have neutralized OPC sensitivity to TRAIL. However, this does not explain why TRAIL was not toxic when administered alone or in combination with OGD.

We found increased expression of DR5 on OPCs *in vivo* after HI by immunofluorescence microscopy. It is likely that this increased expression is induced by microglial and astroglial derived signals, and it remains possible that TRAIL is toxic to OPCs *in vivo*. Additional *in vivo* studies using soluble TRAIL receptors to block TRAIL activity (22) or TRAIL or conditional DR5 knock-out animals may shed light on this issue.

Summarizing these findings, we conclude that TRAIL signaling is important for neuronal cell death after neonatal HI. We have shown that both TRAIL and DR5 are dramatically induced after HI, and our *in vitro* studies show that TRAIL itself is toxic to neurons. Another key finding is that neurons and OPCs have different sensitivities to TRAIL, OGD, and proinflammatory cytokines. The fact that OPCs survive after TRAIL treatment does not rule out the possibility that TRAIL is affecting other biological processes such as differentiation and myelination, critical in the context of the developing brain (40). Further studies are required to fully understand the role of TRAIL signaling in the developing brain after HI injury.

## Footnotes

2 The abbreviations used are:

HI: hypoxia-ischemia
OPC: oligodendrocyte progenitor cell
TRAIL: tumor necrosis factor-related apoptosis-inducing ligand
DR4: death receptor 4
OGD: oxygen and glucose deprivation
dtTRAIL: denatured TRAIL.
